# Obstructed by Two Hearts: Cardiac Tamponade From Mediastinal Lymphoma Requiring Emergent Cesarean Delivery

**DOI:** 10.7759/cureus.95686

**Published:** 2025-10-29

**Authors:** Kendra Robinson, Nolan Jeffery, Hamda Soubagle, Colleen M Achong, Myat M Han

**Affiliations:** 1 Internal Medicine, Medical University of the Americas, Nevis, KNA; 2 Biochemistry, Baylor University, Houston, USA; 3 Internal Medicine, Avera McKennan Hospital and University Health Center, Sioux Falls, USA

**Keywords:** cardiac tamponade, emergency cesarean delivery, mediastinal lymphoma, pneumonia misdiagnosis, primary mediastinal large b-cell lymphoma, respiratory complications

## Abstract

A 33-year-old woman at 37 weeks of gestation presented with progressive dyspnea and cough, initially misdiagnosed as pneumonia. Imaging revealed a large (>15 cm) anterior mediastinal mass compressing the pulmonary arteries and bronchi, along with a significant pericardial effusion. Echocardiography confirmed tamponade physiology. Given the high risk of cardiovascular collapse during labor, a multidisciplinary team, including obstetrics, cardiology, anesthesia, and cardiothoracic surgery, proceeded with emergent cesarean delivery under general anesthesia, followed by subxiphoid pericardial window and excisional biopsy. Postoperative recovery occurred in the intensive care unit, and both the mother and infant remained stable. Pathology confirmed primary mediastinal high-grade B-cell lymphoma, and the patient was promptly started on systemic chemotherapy with DA-REPOCH (dose-adjusted etoposide, prednisone, vincristine (Oncovin), cyclophosphamide, doxorubicin (hydroxydaunorubicin), and rituximab). This case underscores the importance of early recognition, rapid multidisciplinary coordination, and integrated oncologic planning in the management of life-threatening cardiothoracic pathology during pregnancy.

## Introduction

Cancer during pregnancy is rare, occurring in approximately 1 in 1,000 pregnancies, with lymphoma representing the fourth most common malignancy in this population. Non-Hodgkin’s lymphoma (NHL) is a heterogeneous group of lymphoid malignancies that arise from abnormal B, T, or natural killer cells outside the bone marrow. Its incidence has increased significantly since the 1950s, with recent estimates indicating approximately 17 cases per 100,000 women annually [1]. The diagnosis and treatment of malignancy during pregnancy pose complex clinical and ethical challenges, requiring a careful balance between maternal and fetal outcomes.

High-grade B-cell lymphoma (HGBL) is a newly recognized category of aggressive NHL. Previously designated as B-cell lymphoma, unclassifiable, with features intermediate between diffuse large B-cell lymphoma (DLBCL) and Burkitt lymphoma, HGBL was reclassified by the World Health Organization in 2008. These lymphomas are characterized by their blastoid or Burkitt-like morphology and are further subclassified based on genetic rearrangements involving *MYC*, *BCL2*, and/or *BCL6*. When no defining rearrangements are present, the lymphoma is designated as HGBL, not otherwise specified (HGBL-NOS) [2]. These neoplasms are rare, accounting for only 1-2% of NHLs, and are most commonly diagnosed in elderly adults with no sex predominance [3].

Aggressive B-cell lymphomas often present with rapidly enlarging masses and may be associated with systemic B-symptoms, such as fever, night sweats, and unintentional weight loss, although these are not universally present. In rare instances, mediastinal involvement can cause life-threatening complications such as pericardial effusion progressing to cardiac tamponade, where fluid accumulation within the pericardial sac limits cardiac filling and output [4]. Treatment typically mirrors that of other high-grade B-cell lymphomas, using regimens such as dose-adjusted R-EPOCH (rituximab, etoposide, prednisone, vincristine (Oncovin), cyclophosphamide, and doxorubicin (hydroxydaunorubicin)) or modified R-CHOP (rituximab, cyclophosphamide, doxorubicin, vincristine, and prednisone), particularly in cases with MYC rearrangement [5,6]. However, limited data exist to guide therapy for HGBL-NOS, especially in the context of pregnancy.

We present a rare case of primary mediastinal HGBL-NOS diagnosed in a pregnant woman at term, manifesting with non-specific respiratory symptoms and complicated by a large pericardial effusion and signs of cardiac tamponade. This case highlights the diagnostic uncertainty and urgency that can accompany aggressive lymphomas in pregnancy and the need for rapid multidisciplinary intervention.

## Case presentation

**Figure 1:**
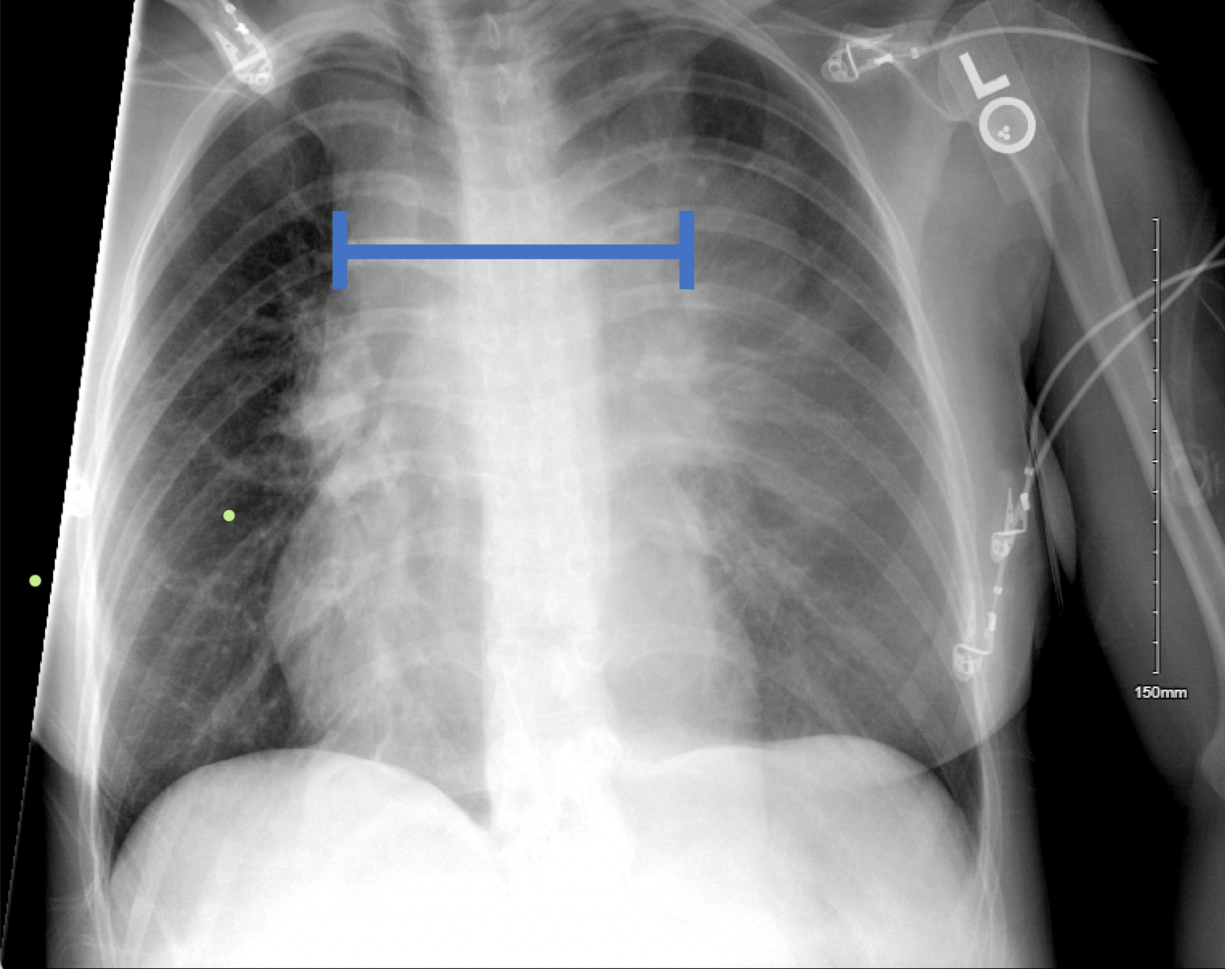
Chest X-ray. Chest X-ray illustrating a widened mediastinum (blue line). This is a pathologic finding and requires further workup.

**Figure 2:**
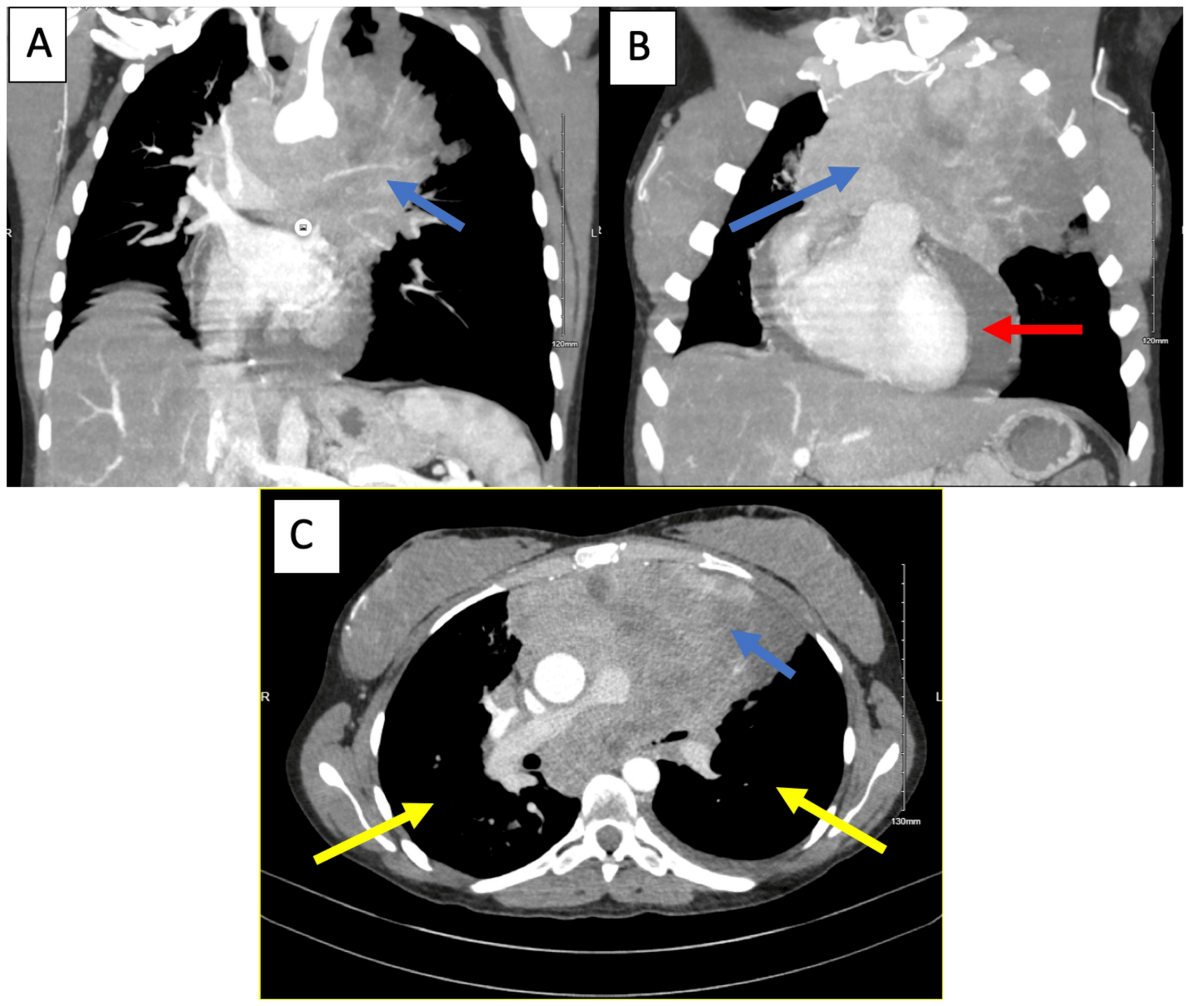
Chest CT. The initial chest CT demonstrates (A, B) >15 cm anterior mediastinal mass with extension into bilateral hilum and posterior mediastinum, moderate to severe pericardial effusion (blue arrows), (B) left pleural effusion (red arrow), and (C) bilateral ground-glass opacities (yellow arrows).

**Figure 3:**
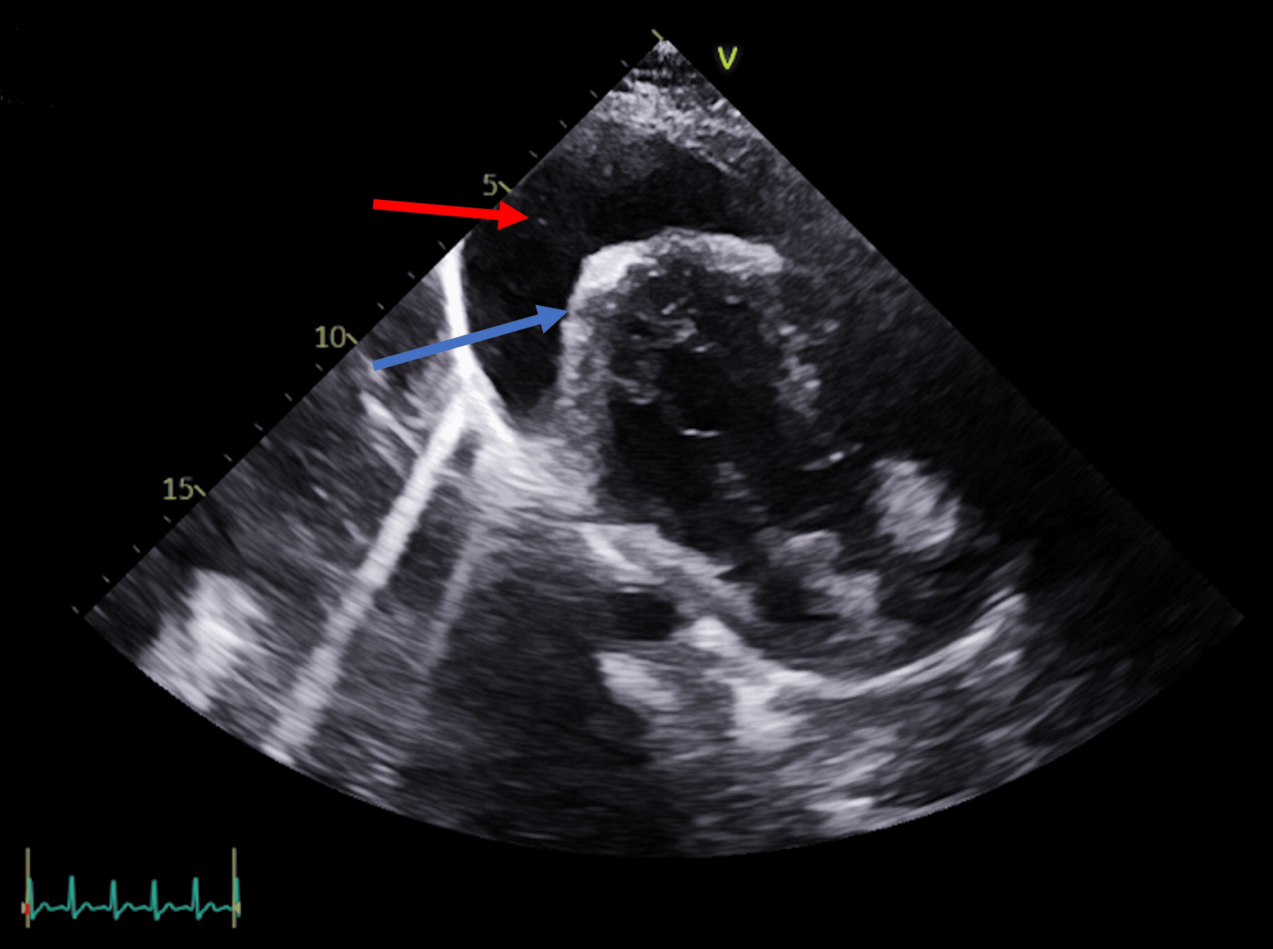
Echocardiogram showing large pericardial effusion. Pericardial effusion (red arrow). Pericardium (blue arrow). Large pericardial effusions, such as this one, can precipitate cardiac tamponade, which compresses the heart and results in decreased cardiac filling, thereby impairing systemic circulation.

A 33-year-old G3P2 woman presented with persistent shortness of breath and a six-week history of cough. Initially diagnosed with pneumonia, she was treated with amoxicillin without symptom relief. A chest X-ray (Figure 1) and CT scan (Figure 2) at 37 weeks’ gestation displayed a widened mediastinum and a >15 cm anterior mediastinal mass encasing the esophagus and trachea, and compressing the pulmonary artery. The mass extended into the bilateral hilum and posterior mediastinum. Bilateral ground-glass opacities were also observed on CT (Figure 2). These findings made the mass inoperable. Multiple enlarged pericardial and left supraclavicular lymph nodes, as well as right lung nodules and renal nodules, raised concern for metastatic disease. The mass exerted significant pressure on the left main pulmonary artery and bronchus, leading to a mild mediastinal shift. An echocardiogram revealed preserved systolic function (ejection fraction of 60-65%), mild tricuspid regurgitation, and an estimated pulmonary artery systolic pressure of ~50 mmHg. A large circumferential pericardial effusion measuring 2.3 cm demonstrated signs of right ventricular diastolic collapse (Figure 3). A mitral respiratory inflow variation of 28% was observed, indicative of physiological tamponade. Epstein-Barr virus testing was conducted, as it correlates with B-cell lymphoma, and the patient tested positive.

The presence of both a mass and a pericardial effusion raised a strong suspicion of malignancy. Given the distended abdomen and the heightened risk of effusion recurrence, pericardiocentesis was considered impractical, leading to the recommendation for a pericardial window procedure. The Obstetrics and Gynecology and Maternal-Fetal Medicine teams advised an immediate delivery, as vaginal delivery was ruled out due to the potential risk of cardiac tamponade decompensation. To minimize maternal and fetal mortality risks, an emergency cesarean section under general anesthesia was recommended, along with a concurrent subxiphoid pericardial window.

The cesarean section was performed, resulting in the delivery of a female neonate with Apgar scores of 2, 8, and 9 at one, five, and ten minutes, respectively. The neonate was admitted to the neonatal intensive care unit for observation. A subxiphoid pericardial window and biopsy evacuated 300 cc of clear pericardial fluid. An excisional biopsy was performed on the anterior mediastinal mass and submitted for pathological evaluation.

Lymphoma was suspected, and while awaiting the pathology report, the patient was initiated on 1 mg/kg of methylprednisolone daily and 150 mg of allopurinol twice daily as prophylaxis against tumor lysis syndrome (TLS) (Table 1). An MRI, with and without contrast, was ordered to evaluate for metastasis, which returned normal results. The pathology report confirmed a diagnosis of primary mediastinal HGBL. Consequently, the patient was promptly started on the EPOCH regimen, scheduled to recur every three weeks. Rituxan was planned to be added at the conclusion of the EPOCH treatment, and allopurinol was continued to prevent TLS. Antiviral acyclovir was also introduced to be administered twice daily.

**Table 1:** Initial laboratory results before treatment. Initial laboratory results before treatment with 1 mg/kg of methylprednisolone daily and 150 mg of allopurinol twice daily as prophylaxis against tumor lysis syndrome. Additionally, hypocalcemia is associated with tumor lysis, as calcium binds to phosphorus released from lysed cells. Calcium is normally an intracellular ion. (L) represents low values. (H) represents high values.

	Initial result	Range	Units
Lactate dehydrogenase	460 (H)	140–271	U/L
Uric acid	4.6	2.3–6.6	mg/dL
Potassium	4.7	3.5–5.1	mmol/L
Calcium	8.4 (L)	8.6–10.3	mg/dL
Phosphorus	4.3	2.5–5	mg/dL

## Discussion

In this case report, we present a notable instance of HGBL-NOS diagnosed in a 33-year-old female at 37 weeks of gestation. The patient presented with shortness of breath, clinically diagnosed as pneumonia versus bronchitis. When the patient failed to improve with antibiotics, CT imaging revealed a large anterior mediastinal mass. A distinguishing feature of this case was the absence of presenting symptoms despite the tumor measuring >15 cm and demonstrating metastatic spread to the supraclavicular lymph nodes, lung nodules, and renal nodules. Ultrasound also revealed an associated large pericardial effusion with physiology indicative of tamponade (Figure 3). The patient was treated with DA-R-EPOCH, consisting of etoposide phosphate, prednisone, vincristine sulfate, cyclophosphamide, doxorubicin hydrochloride, and rituximab.

Previous studies have documented cases of B-cell lymphoma diagnosed in pregnant patients, at gestational ages from 16 to 31 weeks, and maternal ages ranging from 26 to 39 years, consistent with the demographics of the present case. HGBL-NOS itself is uncommon, with a median age at diagnosis of 70 years, making its occurrence in a young pregnant patient particularly unusual. Moreover, the presence of a pericardial effusion, alongside lung and renal nodules, makes this case both complicated and unique.

One of the primary challenges in this case is the coexistence of cancer during pregnancy, a scenario that inherently renders both the fetus and mother immunocompromised. The incidence of malignancies during pregnancy is relatively rare, occurring in approximately 1 in 1,000 pregnancies; however, lymphoma is among the more frequently encountered malignancies in this context, alongside melanoma, breast cancer, cervical cancer, and leukemias [7]. In this instance, malignancy was not immediately considered in the differential diagnosis for a previously healthy female experiencing an uncomplicated pregnancy, especially given the absence of a family history of lymphoma. HGBL-NOS is particularly rare and typically presents in older populations, with a median age of diagnosis around 70 years.

The diagnosis was further complicated by the pregnancy. The patient’s symptoms were attributed to bronchitis vs. pneumonia; pregnancy complicated the diagnostic process of using imaging. The concerns surrounding fetal exposure to radiation posed a significant dilemma; during the initial evaluation, clinicians likely opted for a clinical diagnosis to avoid unnecessary radiation exposure. This decision was also weighed against the potential risk of ionizing radiation exposure to breast tissue, significant given the ongoing glandular proliferation associated with pregnancy, which, although minimal, could increase the future risk of breast cancer and unforeseen complications to the fetus [8]. Thus, the development of a pericardial effusion via ultrasound ultimately warranted more advanced imaging to guide further management. Research has found that cardiac tamponade occurrence has been associated with DLBCL, particularly in immunocompromised patients [9].

In this case, the patient was managed with the DA-R-EPOCH chemotherapy regimen, administered every three weeks. While HGBL-NOS is typically treated using the same protocols as DLBCL, questions remain regarding whether standard DLBCL regimens provide adequate therapeutic efficacy in HGBL-NOS. Ongoing research continues to examine the outcomes of DLBCL-based treatment in this high-grade subtype, which is characterized by more aggressive clinical behavior and poorer prognosis in some cases [2].

The clinical management was further complicated by the patient’s pregnancy, which introduced additional risks and necessitated multidisciplinary coordination. Vaginal delivery was contraindicated due to the increased cardiovascular stress it would impose, potentially exacerbating her hemodynamic instability and risk of cardiac tamponade. Given her high risk for decompensation, the decision was made to proceed with immediate delivery via cesarean section to stabilize maternal condition and allow for oncologic treatment initiation. Following the cesarean delivery, a pericardial window was done to reduce the stress on the heart. A window was chosen to reduce the chances of the recurrence of the pericardial effusion.

Initial emergency department management included consideration of pericardiocentesis versus surgical pericardial window. Due to technical difficulties posed by a distended gravid abdomen and concerns regarding effusion recurrence, the pericardial window was selected as the most appropriate intervention. Steroid therapy was initiated empirically while awaiting final pathology, in recognition of the aggressive nature of the clinical presentation and the need for timely chemotherapy initiation. Neuroimaging with MRI of the brain was also performed to evaluate for central nervous system involvement, which, although uncommon, can occur in high-grade lymphomas and may influence therapeutic decisions.

## Conclusions

This case underscores the diagnostic and therapeutic challenges posed by HGBL-NOS, particularly in the setting of pregnancy. Symptoms of lymphoma, such as fatigue, edema, or dyspnea, may mimic or overlap with normal gestational changes, potentially delaying diagnosis. As such, clinicians should maintain a high index of suspicion in pregnant patients presenting with atypical or rapidly progressive symptoms.
